# Ethical quandaries in spiritual healing and herbal medicine: A critical analysis of the morality of traditional medicine advertising in southern African urban societies

**DOI:** 10.4314/pamj.v10i0.72212

**Published:** 2011-09-21

**Authors:** Mawere Munyaradzi

**Affiliations:** 1 Department of Social Anthropology at the University of Cape Town, South Africa, Universidade Pedagogica, Mozambique

**Keywords:** Keyword Advertising, herbal, spiritual, healing, traditional medicine, morality, southern Africa

## Abstract

This paper critically examines the morality of advertising by practitioners in spiritual healing and herbal medicine heretofore referred to as traditional medicine, in southern African urban societies. While the subject of traditional medicine has been heavily contested in medical studies in the last few decades, the monumental studies on the subject have emphasised the place of traditional medicine in basic health services. Insignificant attention has been devoted to examine the ethical problems associated with traditional medicine advertising. Critical look at the worthiness of some advertising strategies used by practitioners in traditional medicine in launching their products and services on market thus has been largely ignored. Yet, though advertising is key to helping traditional medicine practitioners’ products and services known by prospective customers, this research registers a number of morally negative effects that seem to outweigh the merits that the activity brings to prospective customers. The paper adopts southern African urban societies, and in particular Mozambique, South Africa and Zimbabwe as particular references. The choice of the trio is not accidental, but based on the fact that these countries have in the last few decades been flooded with traditional medicine practitioners/traditional healers from within the continent and from abroad. Most of these practitioners use immoral advertising strategies in communicating to the public the products and services they offer. It is against this background that this paper examines the morality of advertising strategies deployed by practitioners in launching their products and services. To examine the moral worthiness of the advertising strategies used by traditional medical practitioners, I used qualitative analysis of street adverts as well as electronic and print media. From the results obtained through thematic content analysis, the paper concludes that most of the practitioners in traditional medicine lack both business and medical ethics. That said, the paper urges practitioners to seriously consider the morality of their adverts as in most cases they (adverts) do more harm than good. Further to that, the piece recommends the governments of the affected countries to put in place stringent measures to address this mounting problem.

## Introduction

Health has always been a concern for all human societies since the beginning of history. While the subject of traditional medicine has been heavily contested in medical studies in the last few decades, the monumental studies on the subject [1–4] have emphasised the place of traditional medicine in basic health services. In the Western world traditional medicine dates back to ancient Greece and its famous doctors like Hippocrates and Galen. However, although all other civilisations from the ancient world were using plants as natural remedies for their ailments the first documented accounts of the use of herbs as traditional medicine originated in China [5]. In African societies and in Asia in general, though traditional medicine has been used for centuries now, its use seems to have increased in the contemporary times with the advent of diseases like HIV/Aids.

In the African continent, Mozambique, South Africa and Zimbabwe are some of the countries in the fore on the use of traditional medicine. In these countries, socio-economic and political pressures in addition to the prevalence of deadly diseases such as HIV/Aids contribute to the use of traditional medicine. This is to say that the use of traditional medicine has reached an advanced stage in southern Africa in the last few decades mainly through medical traditional practitioners/traditional healers. Having made the same observation, Olapade [6] notes that there has been a global resurgence in traditional medicine in the last ten years probably because of many of the known synthetic drugs in allopathic or Western medicines for the treatment of various ailments are failing or that the causes of these various diseases are developing resistance to the known drugs.

Besides, in southern African societies like Mozambique, South Africa and Zimbabwe traditional medicine healing has been associated with witchcraft, hence viewed with negative and pejorative connotations. This has been chiefly because of Eurocentric paradigms of Africa where the perjured interpretations of Africa have remained grafted on the mental processes and human aspirations of modern Africans thereby robbing them of their intellectual confidence and mental identities with regard to posterity. Unfortunately, many traditional medicine practitioners have stretched this kind of thinking further through their immoral advertising strategies.

However, as previously highlighted the massive socio-cultural, economic and political changes in southern Africa since the attainment of political independence have generated immense pressure on the post colonial states to provide primary health care for all. These changes have brought in renewed socio-economic challenges in the region. Consequently, many people have resorted to traditional medicine; “the role and efficacy of traditional medicine and spiritual services have once again moved to the spotlight” [7]. These courses of events explain the reason why a new crop of traditional medicine practitioners from different countries in the region has emerged claiming to be well-researched, gifted and educated. It has been estimated that in South Africa, for example, between 60% and 80% of the population currently rely on the traditional medical sector as their first contact for advice and treatment of health concerns [8]. Such an increased demand in traditional medicine services in the region (southern Africa) has lured not only practitioners from within the region, but from as far as India and China. Thus, there has been increased competition among traditional medicine practitioners, leading them to employ all advertising tactics and strategies at their disposal in order to lure customers and withstand the fierce competition from other practitioners. Therefore, many of these practitioners have been caught up in ethical quandaries-moral dilemmas- on how they should advertise themselves in a way that would make them more popular than others. From the results obtained from this study, it appears that most of the practitioners have decided to shun “clean advertising” or acceptable moral advertising strategies in favor of “dirty advertising”, that is, immoral advertising strategies. This is chiefly because it has not been easy for most of the practitioners to withstand competition from their counterparts. In the light of this situation, the drive for profit making seems to be overriding many traditional healers’ ought to be morally upright. Thus, the upsurge of immoral business practices in urban southern African societies is testimonial to the unwillingness of the privies to these social ills. So is the prevalence of the so-called myth of amoral business that has invaded the traditional medicine arena.

That said, this paper examines the interface of advertising strategies and morality/ethics in the context of traditional medicine in southern African urban societies. For purposes of this study, the concept morality shall be used interchangeably with ethics. Using qualitative analysis of selected cases of adverts drawn from the aforementioned countries of reference, street corners, electronic and print media, I argue that the persuasive techniques that are employed in advertising by those in traditional medicine often do more harm (to prospective customers) than good. While in business, adverts are meant to truthfully inform the public/prospective customers about the goods and services that are available on the market, those by the practitioners in southern African urban societies normally say the opposite of the reality on the ground.

In view of the foregoing, the piece argues for the integration of business and ethics from two main dimensions namely African communalism, utilitarianism and or consequentialist approach. Using these dimensions, the relationship between business elements like advertising and practitioners in traditional medicine is examined.

Although it is acknowledged in this paper that the answer to medicinal business- moral problems is very difficult to stipulate, the paper suggests that moral teaching, through civic education to the public (potential customers/consumers) and the traditional healers is necessary. Overall, this paper is an attempt to reconstruct and integrate an ethical culture in traditional medicine by demonstrating how the deployment of some advertising techniques by some traditional healers flout the basic principles of both medical and business ethics.

### Herbal medicine, spiritual healing and/or traditional medicine defined

Since I noted in the abstract that the present study critically examines the morality of advertising by practitioners in spiritual healing and herbal medicine, also referred to as traditional medicine, there is need to define herbal medicine and spiritual healing before looking at the history of advertising practices in modern Africa. Herbal medicine and spiritual healing have not been easy concepts to define with precision. However, technical definitions have been offered. According to Australian Journal of Herbalism (http://www.nhaa.org.au/index.php) herbal medicine is medicine made exclusively from plants; it refers to using a plant′s seeds, berries, roots, leaves, bark, or flowers for medicinal purposes [9]. It is the oldest and still the most widely used system of medicine outside of conventional medicine in all cultures in the world today. It is important to note that herbal medicine is also known as botanical medicine or phytomedicine which means using a plant′s seeds, berries, roots, leaves, bark, or flowers for medicinal purposes [10]. As given by the same source, herbal medicine is becoming more main-stream as improvements in analysis and quality control along with advances in clinical research show the value of herbal medicine in the treating and preventing disease. However, scientists are still unsure of what specific ingredient in a particular herb works to treat a condition or illness. This is because the whole herbs contain many ingredients, and they may work together to produce a beneficial effect. Many factors determine how effective an herb will be. For example, the type of environment (climate, bugs, soil quality) in which a plant grew will affect it, as will how and when it was harvested and processed [10].

While herbal medicine is somehow technically easy to define, spiritual healing is not. According to Grayson [11] most people think of spiritual healing as faith healing, but it is not. For him, faith healing has to do with an unquestioned belief in some great God “out there” or some vast power — also “out there.” Sometimes it takes the form of a shaman, a person who is empowered to produce extraordinary, magical effects in the world of the person who believes in him. These aspects explain why the definition of spiritual healing difficult to pin down with precision, therefore contentious. However Grayson (ibid) notes that the underlying principle of spiritual healing is that in the spiritual mind healing process, one does not deal with unquestioned belief in any holy person, religious rite, sacred place or object. Instead, one deals directly with the truth of being which should be perceived metaphysically as part of the underlying Reality of Life. During spiritual healing, the metaphysical inner patterns of consciousness inevitably appear as an outer world of experience, thereby making one conscious of the truth that life belongs to God, or the Great Spirit. It is this realization that enables one to perform spiritual healing.

Critical analysis of definitions of herbal medicine and spiritual healing above shows a broad range of characteristics and elements that make the two interwoven and therefore hardly distinguished from each other. As such, there has been no standard definition for spiritual healing [12] and herbal medicine as both terms can refer to widely varying practices, and there are many ways in which they overlap. It is for this reason and for purposes of this work, that both the term spiritual healing and herbal medicine are used to refer to traditional medicine. That said, we also need to define traditional medicine. World Health Organization's (WHO) definition of traditional medicine which says: *“Traditional medicine includes diverse health practices, approaches, knowledge and beliefs incorporating plant, animal and/or mineral based medicines, spiritual therapies, manual techniques and exercises applied singularly or in combination to maintain well-being, as well as to treat, diagnose or prevent illness* [13]*. ”*

As has been highlighted above, in Africa and as elsewhere, herbal medicine and spiritual healing are both embodied within traditional medicine which in itself is the total sum of all knowledge and practices used by traditional healers in diagnosis, prevention and elimination of physical, mental or societal imbalance. This knowledge or practices can be explicable or not. The underlying fact is the knowledge/practices are handed down spontaneously from one generation to another, normally through the aid of a traditional medicine practitioner/traditional healer. This can be through orature, literature, observation or even through mystical ways. I identify with Pretorius who defines traditional medicine practitioner/traditional healer is defined as: *“someone who is recognised by the community in which he lives as competent to provide health care by using vegetable, animal substances and certain other methods based on social, cultural and religious backgrounds as well as the prevailing knowledge, attitudes and beliefs regarding physical, mental and social-well being and the causation of disease and disability in the community* [7]*.”*

As already alluded to above, there are two main kinds of traditional healers that can be identified. These are: herbalists and diviners (diagnostician or divine mediums respectively) [8]. It should however be noted that different ethnic groups have their own legends about the origins of traditional medicine in their own society.

## Background to advertising in traditional medicine in Africa

Sub-Saharan Africa has a long tradition of traditional medicine advertising. In the past, advertising was normally done orally, that is, by word of mouth. This was done by the practitioners themselves and or by clients and neighbors. As such, advertising of traditional medicine though now more common than ever is not a new and unique phenomenon to southern Africa, but is resonant as in other countries in the region and beyond.

While the subject of traditional medicine has been heavily contested in medical studies in the last few decades in sub-Saharan countries like abovementioned, the monumental studies on these subjects [1–3] have emphasised the place of traditional medicine in basic health service. Adegoju [4] though have criticized the abovementioned scholars has fallen in the same trap as he takes a linguistic stance and focuses solely on the rhetorical style used by herbal medical practitioners in Southwestern Nigeria in launching their products. As such, all the aforementioned scholars, among others, have devoted insignificant or no attention to examining the moral worthiness of some advertising strategies used by practitioners in traditional medicine when launching their products and services on market. Yet, though advertising is key to helping practitioners’ products and services known by prospective customers, this research establishes a number of morally negative effects that seem to outweigh the merits that the activity brings to society.

In urban societies of Mozambique, South Africa and Zimbabwe, the linguistic advertising strategies employed by traditional medicine practitioners/traditional healers have mired the advertising of herbal and spiritual healing services with a plethora of controversies. The controversies are further compounded by the nature of advertising discourse itself which many business ethicists [14, 15] believe is psychologically coercing, misinforming, cunning and void of ethical principles.

In addition, though Mozambique, South Africa and Zimbabwe are some of the countries with a very long history of the development and use of herbal medicine and spiritual healing techniques, the national governments of these countries have always suppressed traditional medicine's advertising and use in favor of conventional/scientific medicine. This is because traditional medicine has always been associated with witchcraft. It should be noted that in these countries colonial systems’ health care legacy remains a problem with their bias towards allopathic health care. In fact the debate on the legitimacy of traditional healers has been highly contentious because of the complex political, legal, cultural and social ramifications of the practice. From a politico-historical perspective, the colonial government regimes perplexed by failure to establish and provide proof and distinction between traditional healing and witchcraft in legal battles outlawed both (traditional healing and witchcraft) for legal and administrative convenience. Unsurprisingly, the post southern African governments haunted by the same dilemma retained the colonial legislations like the Witchcraft Suppression Act where formal courts of law were under obligation not to recognize both traditional healing practices and witchcraft unless the plaintiff provided substantial evidence linking the defendant to the practice. In Zimbabwe, for example, this saw the enactment of the Zimbabwe's Witchcraft Suppression Act (ZWSA) (Chapter 73) which considers as illegal an activity that has to do with supernatural forces or charm meant to inflict injury, misfortunes, illness or death. Likewise, South Africa saw the enactment of restrictive legislations such as the Witchcraft Suppression Act in 1957 and the Witchcraft Suppression Amendment Act of 1970. In Mozambique, though no known laws were enacted against witchcraft or activities related to it, traditional healing until recently was equated to witchcraft. Worse still and in line with the misplaced thinking against traditional medicine practitioners (as elsewhere in the region), there have been numerous media reports about people who have been killed for *muti* (charm used in witchcraft) purposes under the instruction of traditional practitioners. As Mutungi [16] puts it: *“Witchcraft beliefs embrace a wide range of ideas, practices, and motivations, but in their various forms they usually share the idea that the power to inflict injury and benefit could be exercised through unobservable, supernatural means.”*

In southern Africa, the word witchcraft as the concept of traditional medicine has therefore earned negative and pejorative labels. This has been chiefly because of Eurocentric paradigms of Africa where the perjured interpretations of Africa have remained grafted on the mental processes and human aspirations of modern Africans thereby robbing them of their intellectual confidence and mental identities with regard to posterity. Unfortunately, many traditional medicine practitioners, especially those in contemporary urban societies have stretched further and authenticated this misplaced thinking through their immoral advertising strategies.

In the aforementioned countries, and sub-Sahara in general, the massive socio-cultural, economic and political changes since the attainment of political independence and a general renewed challenge to the post colonial state to provide primary health care for all brought cultural crunches and challenges in the region. They (southern African states) had to resort to herbal medicine and spiritual healing, hence making “the role and efficacy of traditional medicine services once again moved to the spotlight” [7]. These courses of events explain the reason why a new crop of traditional medicine practitioners from different countries in the region has emerged claiming, through advertising, to be well-researched, gifted and educated. It has been estimated that between 60% and 80% of South Africans currently rely on the traditional medical sector as their first contact for advice and treatment of health concerns [8].

Due to socio-economic and political pressures in the southern African region, there has been increased demand in traditional medicine services luring not only practitioners from within the region, but from as far as India and China. As a matter of consequence, there has been increased competition among traditional medicine practitioners, leading them to employ all tactics and strategies at their disposal. These include immoral advertising strategies such as false testimonials and misinformation in order to lure customers and withstand the fierce competition from other service providers. As previously highlighted, most if not all of these practitioners have therefore tended to shun “clean advertising” in favor of “dirty advertising” in order to withstand competition from their counterparts. As never been before (in the past) profit making has emerged to challenge and override many traditional healers’ ought to be morally upright. Thus, the upsurge of immoral business practices, through immoral advertising strategies, in contemporary southern African urban societies has reached an alarming level. So is the prevalence of the so-called myth of amoral business that has invaded the traditional medicine arena. Most traditional healers, for example, have generally accepted the myth of amoral business- “a belief that business and ethics do not mix” [15] -they are mortal enemies. They have done so by employing unscrupulous advertising tactics/strategies. Some of the unethical advertising practices that have unapologetically infiltrated the southern African herbal medicine and spiritual healing arenas are misinforming, coercing, cunning, deception and other such problems associated with advertising. These practices have manifested at an increasingly alarming rate invoking the anti- advertising partisans and the consumers in general arguing for the banning of advertising of traditional medicine by traditional healers.

What most if not all of these practitioners have forgotten or failed to realize is the fact that “dirty advertising”-immoral advertising strategies- void of acceptable ethical practices can dramatically compromise their reputation in the society. The deployment of “dirty advertising” has therefore presented a serious moral test case for traditional healers in traditional medicine in southern Africa and beyond.

## Research questions and methodological issues

The present study seeks to address the questions: Which moral wrongs are being committed in the name of advertising by practitioners in traditional medicine? How best can we right the wrongs committed by practitioners in traditional medicine? As a researcher on medical issues on Africa in general and southern African region in particular, I have come to the realization that most researchers on traditional medicine [1–3] have emphasised the place of herbal medicine in basic health service. Adegoju [4] taking a linguistic stance, emphasises on the rhetorical style used by traditional medicine practitioners in Southwestern Nigeria in launching their products. All these scholars, among others, are guilt of devoting insignificant or no attention to examining the moral worthiness of some advertising strategies being used by practitioners in traditional medicine when launching their products and services on market. The history of traditional medicine thus makes a sorry reading with its failure to document, by default or otherwise, the moral wrongs committed in the name of advertising by practitioners in traditional medicine. The consequence is that these moral wrongs are perpetuated in societies thereby making the society suffering undeservingly; hence the need for a study as this.

As part of my research design, I relied on qualitative analysis of data sampled from both the electronic and print media. For the electronic media, I focused on jingles on television and radio in the three countries previously referred to. In addition to the jingles, I focused on the thematic content of the specially sponsored programmes where the advertisers buy airtime that covers at least ten minutes slot or more. Such programmes normally involve interviews where the advertisers tell the audiences what they offer and sometimes the advertisers answer questions from the audiences. The jingles and sponsored programmes are drawn from radio and television stations based in the aforementioned cities. Jingles and sponsored programmes from these different cities were considered in order to come up with an unbiased view of adverts by most spiritual and herbal practitioners in southern African region. One striking observation on the advertisers sampled said more than what their medicines practically cure. Others directly or indirectly undermined conventional medicines thereby widening the gap between scientific medicine and traditional medicine.

The data sampled from print media were adverts and select paid by traditional medicine advertisers, mainly to newspapers, in the countries under study, that is, Mozambique, South Africa and Zimbabwe. It is important to note, however, that there is an overlap between adverts in electronic and print media.

In both electronic and print media, diseases and conditions commonly advertised by practitioners in traditional medicine were asthma, tuberculosis, matrimonial or love related problems, misfortune related problems, sexually transmitted diseases, sterility, and low sperm count, among others.

To supplement the field observation information obtained from electronic and print media, the researcher used observation in the big cities of Mozambique, South Africa and Zimbabwe. The cities observed were Maputo, Beira (Mozambique), Johannesburg, Cape Town (South Africa) and Harare (Zimbabwe). A striking observation about the streets of these cities, where the researcher lived, studied and worked for some years, is the amount of pavement advertisements of traditional medicine services, mainly in the form of leaflets at strategic locations such as road sides, street corners, markets and motor parks where the attention of passers-by can easily be attracted. Using observation data collection procedure the researcher observed 45 pavement advertisements (6 in Beira; 9 in Maputo; 6 in Johannesburg; 9 in Cape Town and 15 in Harare) by different traditional medicine practitioners. The field observation, a method which the researcher adopted from his anthropological studies was used as one of the major collection tools to ascertain the project location and what really is happening on the ground. Observation allows the researcher to have access to first hand information that s/he can observe and record in person. Using this method, the researcher observed that to reach their prospective customers, the advertisers issue out to passers or stick on corner posts leaflets detailing out the title(s), name(s), contact details and place of origin of the traditional healer and the available treatment services for various health ailments and socio-cultural and financial problems. This type of advertising is very aggressive and reaches out to the people directly.

## Common advertising moral wrongs in medicine: Promoting good moral principles in traditional medicine advertising in southern African societies

Business in all spheres, medical fraternity included, is bound by some precepts that all practitioners are obliged to observe. According to Fieser [17] businesses have moral obligations beyond what the law sometimes requires. This is to say “business is supposed to be unscrupulous and driven by the sole need for personal success. But it should consider the customers’ values, interests and needs” [18]. This is contrary to what is transpiring in the advertisement of traditional medicine in southern African urban societies. Instead of what Fieser [15, 17] propose above, advertisement of herbal medicine and spiritual healing services is characterized by a plethora of moral wrongs. These range from psychological coercion, misinforming, exaggerated competence, false guarantees, and false testimonials to the use of rhetoric. Such practices flout and dramatically compromise the basic principles of both business and medical ethics and traditional healers’ reputation in the society. In what follows, a detailed account of advertising moral wrongs abound in southern African societies is presented. Suggestions to deal with them are also given in the account.

### Exaggerated Competence

The advertisement of traditional medicine in southern African societies has been characterised by the moral wrong of exaggerated competence. This is whereby the healer claims to cure diseases or heal problems of all kinds when in reality s/he doesn't have the power or knowledge to do so. This makes exaggerated competence a persuasive strategy that is employed in advertising traditional medicine to raise the healer's profile, credibility and competence. In such immoral advertising strategies, the advertisers pretend to be credible by displaying practical intelligence and in-depth knowledge of medicines and/or spiritual powers. The advertisers normally propagate this credibility in the eyes of prospective customers by clearly outlining the clinical symptoms of diseases, with the view of showing the public that they have thorough knowledge of pathology and could diagnose the patient's diseases even before interacting with them. Below are some of the adverts sampled in southern African urban societies during this study:

1. Abnormal Ejaculation?: Are you a “1-minute man” get ejaculatory powder for you not to sperm quick that your women in stable relationship.
2. Diminished libido/sex drive?: Libido enhancer to give you strong urge in stable relationships.
3. Erectile dysfunction?: This may be due to aging, diabetes, relationship stress hormones or physical problems. A new Herbal Chinese mixture it takes 20 min before sex to give you rock hard erection permanently.
4. Penis Pro-Enlarger: Have you failed from pills, creams, surgeries, pumps and false promises come try the new Chinese remedy and gel rubbed 2 times daily for 1 week to the big cock you ever desired.
5. After fifteen years of thorough research, Prof. Dr. J.J. Diriko finally came to a breakthrough, now introducing (MASAI GEL) for the first time in Mozambique, especially for both men and women. It is a herbal gel from the MASAI land mainly intended to enlarge the penis both in girth and length. In women the gel is prepared in such a manner that it causes the vagina to shrink thereby making a woman appearing a virgin in her late fifties.

The thematic contents of all these adverts undermine the strength of conventional medicine by emphasizing exaggerated competence of the Chinese herbs over scientific medicine. By providing a false detailed background information about oneself as is in the case of “Professor Dr. Diriko” the practitioner obviously intend to magnify his/her credibility. One would think that the healer is a well educated, thorough and highly experienced person when in actual fact he has never attained any Doctoral degree, worse still Professorship. Also, making reference to the countries from which the mixtures, creams, gel and powders are sourced such as China, the advertisers make a gaze towards convincing prospective customers that their medicines are authentic as they come from countries which are well-known for producing effective herbal medicines. All these claims tend to underscore the herbal practitioners’ insatiable quest for acceptability and credibility directed towards convincing or winning the minds of prospective customers. As such, adverts of this nature result in psychological coercion on the part of prospective customers who sometimes will have exhausted the use of scientific medicine.

What remains a truism is that no evidence can be practically made available to support such claims as those made above. In other words, no empirical or scientific evidence to prove that the advertised treatment will result in permanent healing without side effects or allergies is made available to prospective customers. Worse still, there is no evidence for the audience to prove that the healer can cure a myriad of diseases and conditions. It is in the face of this uneasiness that one can critically questions the moral acceptability of such claims. This paper therefore advances the argument that by making exaggerated competence, the healer does not only transgress against the moral precept of advertisement which stresses truth, but compromise his/her moral legitimacy in the society. Also, the advert violates Gricean's [19] maxim of quality, which states that in conversation (and by extension in advertising discourse) one should not lie or make unsupported claims. Hence, affected people are encouraged to take their courage to denounce such adverts. This will serve as a moral whip to discipline and discourage advertisers of traditional medicine from engaging in such immoral advertising strategies as exaggerated competence.

### False Guarantees

From the sampled data, it was also revealed through qualitative analysis of some adverts that another advertising strategy being employed by advertisers of traditional medicine and spiritual healing is false guarantee. This advertising wrong is a persuasive technique which is meant to induce confidence in the potential customers most of whom are likely to have experienced the technique in the orthodox commercial world. It was observed that in southern African urban societies, false guarantees come in the form of false promises to prospective customers. The promises obtained from sampled data were:

1. Get back your lost property, relative or lover is less than two days after treatment
2. Call and book an appointment before you come in, the Dr is always busy
3. A distinguished miracle teller who tells who tells you all about your problems before you say anything and heals diseases of all kind
4. Just come in and do away with all your problems immediate after treatment. 100% guaranteed permanent results
5. Excellent herbs with no side effects for all STDs, itching vagina, womb cleaning
6. ABORTION: Same day, 100% safe and pain free guaranteed. Call Dr. Donald

The above guarantees raise a lot of questions, especially to critical thinkers though they (guarantees) are likely to convince prospective customers haunted by some of the problems outlined. The short time frame (of two days) guarantee, 100% guaranteed permanent results and promises to meet a distinguished miracle teller and restorer of lost properties are all false guarantees that have the potential to psychologically compel prospective customers to come in their numbers. However, on subjecting all these claims on hyperbole literary analysis one may realise that there is no authenticity in them; they are just hyperbole exaggerations used in the name of advertising to lure prospective customers by evoking their feelings. Hyperbole refers to a case where the speaker's description is stronger than is warranted by the state of affairs described [20]. It is from this understanding that I advance the argument that adverts by healers can only be morally justifiable in as long as they uphold truth and abandon the use of false guarantees and other such advertising gimmicks. In this view, I identify with Harris and Seldon [21] who understand advertising as “a form of communication designed to spread accurate information to the public with the view of promoting marketable goods and services”.

### Misinformation

It is a generally well known adage that “lies when documented resemble truths”. Having realised the impact of ‘well cooked’ and documented lies, most southern African advertisers in traditional medicine document eye catching inscriptions, catchy rhetorical titles and exaggerated achievements to win the hearts of their targeted audiences. Most of the titles sampled from southern African urban street corners, electronic and print media were: Prof Dr; Mama; Dr of the year; Best Prophet of all times; Magic man from the Pacific; Expert prince from Indian ocean; The Proud Winner of sub-Saharan Spiritual Healers Award; A well educated, thorough going researcher; A distinguished miracle teller; A specialist of all spiritual problems; and the 2011 African herbalist winner.

Such rhetorical titles are used to create credibility and acceptance by the targeted audience, that is, prospective customers. By using such titles as above, the traditional medicine practitioners attempt to draw parallels between themselves and competent practitioners in conventional medicine who can be entrusted with human life. This confers legitimacy on the healers as the titles suggest that the bearer is competent, rigorous, educated and with formal training in the diagnosis and treatment of health-related ailments, usually in medical schools. What makes their claims dubious, especially to critics is however the fact that unlike mainstream medical doctors who indicate their educational qualifications and the institutions from which they acquired them, practitioners under study don't do the same. This makes people understand that they are being deliberately misinformed. Yet, in an advertising pursuit, misinformation is a moral transgression against the public. Denouncing the immorality of misinformation, Norris [22] argues that “the advertiser should always tell the whole truth about the product he wants to sell and should judge the message not by what it says but by what the reader is most likely to think it says”. The advertiser thus, should not manipulate, misinform or influence the prospective consumer deliberately or otherwise. The consumer should decide on his/her own without any physical or psychological coercion.

### False Testimonials and claims of sources of herbs

The last advertising gimmick sampled in southern African urban societies during this study was the use of false testimonials and claims of sources of herbs. Testimonies by people who claim to have been healed by the practitioners were observed in contents of street corner adverts, electronic and print media. Far reaching sources of herbs were also given. The motivation behind the deployment of testimonies and mentioning far reaching sources of herbs is to create legitimacy, credibility and to instil confidence in prospective customers. It was observed that the people who give these testimonies normally provide their personal names and most of them were from low density suburbs. This was possibly meant to create the impression that the ‘poor’ prospective customers can also end up living in low density suburbs if they visit the practitioner. The sources of the herbs were also mainly under oceans/seas, famous mountains and sacred places. The strategy here thus is twofold: to make prospective customers believe that their problems can really be solved and that their lives can be improved just like those in the low density suburbs. Below are examples of testimonies and sources of herbs from the sampled data:

1. Having consulted several traditional healers but all disappointing me, I went to Dr. Diriko who helped me with magic stick. I now solved my financial problems. (Tatenda Mariyawanda, Borrowdale: Harare)
2. My penis was small. I was shy to propose love to women! I was scared they will laugh at me. I used Chinese herb remedies and in less than 5 days my penis grew to the size I wanted (Peter Jones, Centurion: Pretoria)
3. I always wanted to start my own business but in vain. When I met Dr. Pedro Domingos I picked a bundle of one thousand meticais by the roadside and started my business right away. I am a successful business man in T3 (Paulo Emanuel, Maputo)
4. Dr. Abu Dimao with magical herbs from the Indian Ocean, Mount Kilimanjaro and the Kalahari Desert
5. Mama Dorina with spiritual powers and Chinese, Indian, African, American and Arabic herbs that heal over 100 diseases

It should be noted that the claims of sources of herbs that are sacred, far and the use of testimonies with names of people healed are just but false. One would concur that if these claims and testimonies were true, nothing bad would be said about them. What raises concern is that from the sampled data, it was revealed that most of the names in the testimonies are inexistent. This was credibly true as during the study, the researcher never met any one of the people who made the testimonies; none of them ever came to the fore to show up himself/herself in the public. This has rendered all the sampled testimonies a gimmick and empty diplomacy deployed to engender confidence in prospective customers by raising their hopes. This advertising technique is well documented in the general advertising discourse and is known for invoking a propaganda technique called ‘bandwagon’. Bandwagon is an attempt to persuade the targeted audience to join in and take the course of action that everyone else in a similar predicament is taking regardless of one's financial, health and social condition. The key aspect of testimonies is that they show prospective customers the benefits that other people are getting from healers.

The claim of supremacy over other healers or herbalists by practitioners under study is another false type of false testimony with the potential to make prospective customers believe that the healer in question is better than the rest. In philosophy circles, in particular logic, this technique is known as *fallacy-argumentum ad hominem*. This is a situation whereby people try to win arguments by saying derogatory and negative things about their opponents as is demonstrated in the adverts i) and ii) above. In advertising, this technique though immoral is normally used to out-compete one's counterparts, in this case, other spiritual and herbal medicine practitioners. It is in this light that I argue against false testimonials and false claims of sources of herbs used by most of the practitioners in traditional medicine in southern African urban societies.

## Conclusion

This study has revealed that in contemporary southern African urban societies, advertising is used in many realms like medical fraternity. However, like in the business world, advertising in traditional medicine in southern Africa has met with a number of ethical quandaries and/or challenges. As revealed by the sampled data, the challenges met by traditional medicine have been largely a result of unethical techniques employed by traditional medical practitioners to advertise their products and services. It is in this light that I have argued that the ‘diabolic’ stance and derogatory connotations about the practice of traditional healing adopted in literature partly stems from an acoustic understanding of the practice and unethical advertising principles deployed by the practitioners themselves. This is compounded by the limitations of expert science to explicate the cosmology of the ‘world beyond’, and the conflation of witchcraft with traditional healing [23]. This study has therefore demonstrated that the mounting incidences of unethical and morally unacceptable advertising strategies mire the whole practice of traditional healing with controversies. These incidences have proven beyond reasonable doubt that most traditional healers in southern African urban societies lack both business and medical ethics, hence the need for the national governments of countries concerned to work in cohorts with some independent organisations to empower and provide civic education to both the traditional healers and the public in general.

Most importantly, I have argued that ethics and its influence in business in general and medical fraternity in particular should not be underestimated. I underscored that any business, be it in medicine or elsewhere, can only gain credence and acceptance in society if it regards ethics. The merit of this study therefore lies in its quest to see to it that practitioners in spiritual healing and herbal medicine uphold ethical principles in ways that illuminate understanding of their practices.
